# Hirschprung's disease and postpartum trauma leading to fecal incontinence: Why? How?

**DOI:** 10.1590/1806-9282.20240827

**Published:** 2024-09-13

**Authors:** Milan Tesar, Ilker Sengul, Ivana Mrazkova, Dmytro Klymenko, Demet Sengul, Lubomir Martinek, Anton Pelikan, Olga Szabova, Jan Kümmel, Jan Krhut, José Maria Soares

**Affiliations:** 1 University Hospital Ostrava, Department of Surgery – Ostrava, Czech Republic.; 2 University of Ostrava, Faculty of Medicine, Department of Surgical Studies – Ostrava, Czech Republic.; 3 Giresun University, Faculty of Medicine, Division of Endocrine Surgery – Giresun, Turkey.; 4 Giresun University, Faculty of Medicine, Department of General Surgery – Giresun, Turkey.; 5 Giresun University, Faculty of Medicine, Department of Pathology – Giresun, Turkey.; 6 Tomas Bata University in Zlín, Faculty of Humanities, Department of Health Care Sciences – Zlín, Czech Republic.; 7 University Hospital Ostrava, Department of Gynaecology – Ostrava, Czech Republic.; 8 University Hospital Ostrava, Department of Urology – Ostrava, Czech Republic.; 9 Universidade de São Paulo, Faculdade de Medicina, Hospital das Clínicas, Departamento de Obstetrícia e Ginecologia, Disciplina de Ginecologia, Laboratório de Ginecologia Estrutural e Molecular – São Paulo (SP), Brazil.

## INTRODUCTION

### Hirschsprung's disease

One of the most common congenital anomalies affecting the aboral parts of the gastrointestinal tract is Hirschsprung's disease. It is characterized by the absence of nerve ganglion cells in the Meissner (submucosal) and Auerbach (muscular) nerve plexuses^1^. This rare disease (1 in 5,000 live births) leads to permanent spasms of the aganglionic segment and causes progressive dilatation of the colon above the affected intestinal segment^2,3^. The most common clinical symptoms are abdominal distension (>90%), vomiting (>85%), and delayed passage of meconium—typically after 24 h after birth (>60%)^4^. Of note, classical rectal examination or insertion of a rectal tube usually leads to explosive evacuation of gas and stool, which typically have a foul odor. The essential aspects for diagnosing Hirschsprung's disease are medical history, clinical course, radiological examination, and histopathological examination of seromuscular samples obtained by rectal biopsy^5^. Currently, the only rational solution to manage this disease is surgical therapy.

### Postpartum anal sphincter injury

In the field of fecal incontinence, the focus for gynecologists and surgeons is on caring for women with postpartum anal sphincter injury and women with anorectal dysfunction due to pelvic floor static disorder (pelvic organ prolapse). Obstetrical anal sphincter injuries (OASIS) is a fundamental comparative indicator of maternal morbidity, which leads to significant comorbidities, including perineal pain in the early postpartum period and, in the long term, dyspareunia, sexual dysfunction, and anal incontinence. Birth trauma, per se, includes damage to the anatomical complex of the anal sphincter and pudendal neuropathy. Despite surgical intervention, some women have residual sphincter defects. The occurrence of OASIS has been reported in^4-6^ 6% of all vaginal deliveries, with a higher rate in operative vaginal deliveries and precipitous births^6^. The incidence of OASIS depends on many risk factors^7^. The protective nature of episiotomy has not been proven nor routinely used. Episiotomy is indicated in cases of impending fetal hypoxia to expedite delivery or in operative delivery^8^. The type of third-degree tear impacts the severity of complications. Injury to external and internal sphincters has the same symptomatology as a complete injury, including rectal mucosa. Untreated rectal mucosa damage can lead to the development of a rectovaginal fistula^9^.

### Fecal incontinence

Fecal incontinence (FI) prevalence increases with age but often goes unreported to healthcare providers. It is associated with an increased incidence of various skin conditions and inflammatory diseases, including numerous urinary tract infections. Patients with FI are left only with incontinence products (such as pads and adult diapers) after the failure of all available conservative methods. If conservative methods fail to improve symptoms, surgical options are considered, such as sphincter reconstruction, sacral nerve stimulation, and anal sphincter augmentation^10^.

### Sacral neuromodulation

Sacral nerve stimulation or sacral neuromodulation (SNM), is an effective treatment for a range of colorectal and urological conditions, such as urinary retention, fecal incontinence, constipation, and other bowel dysfunctions. Anal sphincter function can be improved by placing a sacral or percutaneous tibial nerve stimulation. In recent decades, SNM has significantly advanced the treatment of fecal incontinence, whose technique involves two surgical procedures^11^. Although the exact mechanism of SNM remains unknown, the results are highly promising, with more than two-thirds of patients experiencing more than a 50% improvement during the trial phase, leading to permanent implantation of the modulator. After permanent implantation, 86–87% of patients report more than 50% improvement, and approximately 40% achieve complete control, with effects lasting longer than 3–5 years. The number of complications is relatively low, the most common being infections and electrode displacement, occurring in 3 and 12%, respectively^12^.

### Presentation

Herein, we also would like to mention the case outlining several issues that have gradually affected a single patient. This report provides a fundamental analysis of these nosological units, including the possibility of surgical solutions for each situation. A 26-year-old female with asthenic habitus (BMI 18.8) was referred to the proctology clinic for fecal incontinence from the urogynecology clinic. She underwent a Swenson's procedure at the age of 6 months, very likely with rectal resection for the classic form of Hirschsprung's disease.

Histopathology of a rectal biopsy confirmed Hirschsprung's disease. The irrigography confirmed sigmoid dilation with a circular cut in the rectal area and a narrow rectal ampulla. A scar from a midline laparotomy on the abdominal wall, extending from the xiphoid process to the pubic symphysis, was recognized. She has struggled with frequent bowel movements throughout her life, approximately five to eight times a day, but was fully continent. At the age of 24 years, she completed her first physiological pregnancy with a spontaneous vertex delivery, and a cesarean section was contraindicated by gynecologists due to the uncertain extent of childhood surgery and the risk of perioperative complications. The first and second delivery stages progressed rapidly, with a physiological fetus weighing 3,470 g. A perineal rupture occurred during delivery, classified as grade IIIa according to available documentation. The injury was treated in the delivery room under local anesthesia with 1% Mesocain, and the suture was performed using Novosyn quick 2/0 suture material. A year and 8 months after giving birth, she visited a urogynecology clinic due to incontinence issues. The frequency of bowel movements was 15–30 times a day, in small amounts, with an urgency of less than 1 min. She suffered from soiling and could not retain gas. On the gynecological examination, the sphincter in the perineal area was poorly palpable. An ultrasound of the perineum did not identify the internal sphincter; the external sphincter appeared intact, but the findings were inconclusive.

**Figure 1:**
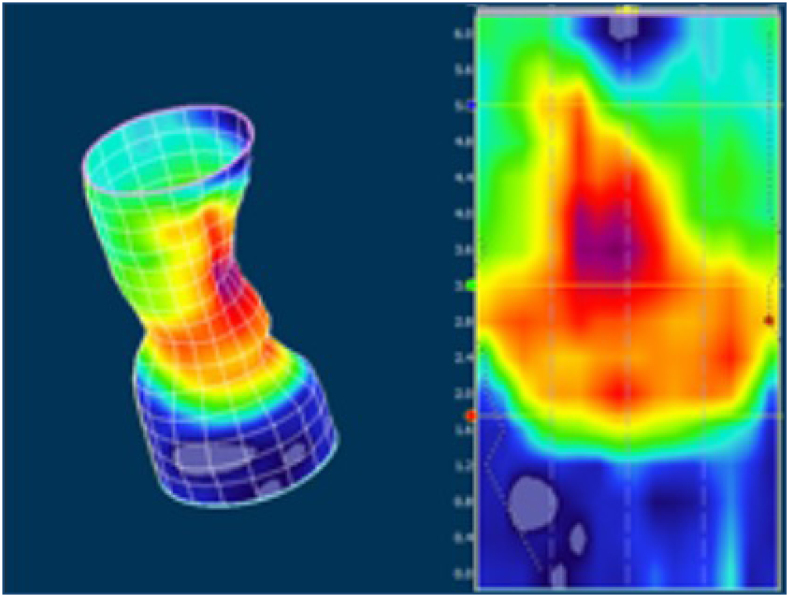
An intraoperative photography; the overlap plastic procedure.

**Figure 2:**
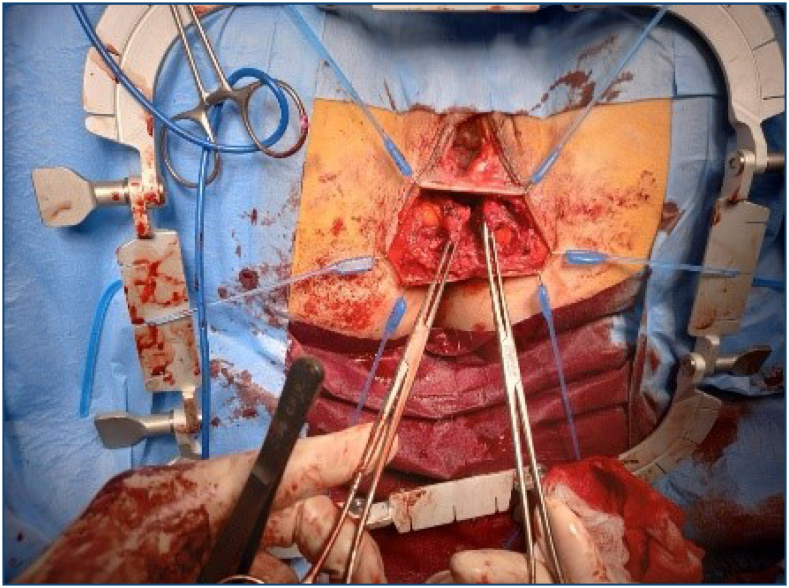
A 3D manometric model of the sphincter.

During an examination at the Proctology Clinic, a gaping anus was found with a palpable lesion of the sphincters over an area of approximately 30°. Their contraction was only present on the lateral sides and dorsally, and the perineum was thinned. The sphincter lesion was confirmed by endosonography, but the internal sphincter was again not identified. Her colonoscopy was negative, but the 3D manometry confirmed a lesion in the ventral portion of the sphincters (Figure 1). She was indicated for sphincter reconstruction using the overlap method for fecal incontinence, with a Wexner score of 16. The surgery was performed under general anesthesia 26 months after delivery without perioperative complications. A sphincteroplasty using the overlap method and an anterior levatoroplasty were performed. For this purpose, a transverse incision in the perineal area was made after placing the Lone Star retractor to access the sphincters, and an overlap plasty was done in three points using PDS 3/0 suture material, followed by an anterior levatoroplasty (Figure 2). A Redon drain was inserted, and the skin was sutured perpendicularly. She was discharged on the fifth postoperative day.

A month after the surgery, the local examination was unremarkable, and the anus was spontaneously closed and readily patent for a finger, with palpable sphincter tone throughout. However, despite short-term postoperative improvement, bowel movements were again very frequent, up to 15 times a day, with questionable bowel control. She was referred for pelvic floor rehabilitation, biofeedback, and sphincter electrostimulation 6 weeks after the surgery. Loperamide was prescribed to slow down bowel movements.

Four months after the sphincter surgery, she still had frequent bowel movements—10 times a day, with no significant improvement in continence. The local finding was calm; it was healed, and the anus was closed, with a palpable thinning in this area. She underwent endosonography, which revealed a 5 mm defect in the external sphincter area, with the internal sphincter not identified again. An anal manometry was performed, revealing a resting pressure of 22 mmHg, a maximum squeeze pressure of 110 mmHg, and a weakly evocable rectoanal inhibitory reflex with reduced rectal capacity. The neurological examination showed no pathological findings, and she was indicated for SNM.

**Figure 3:**
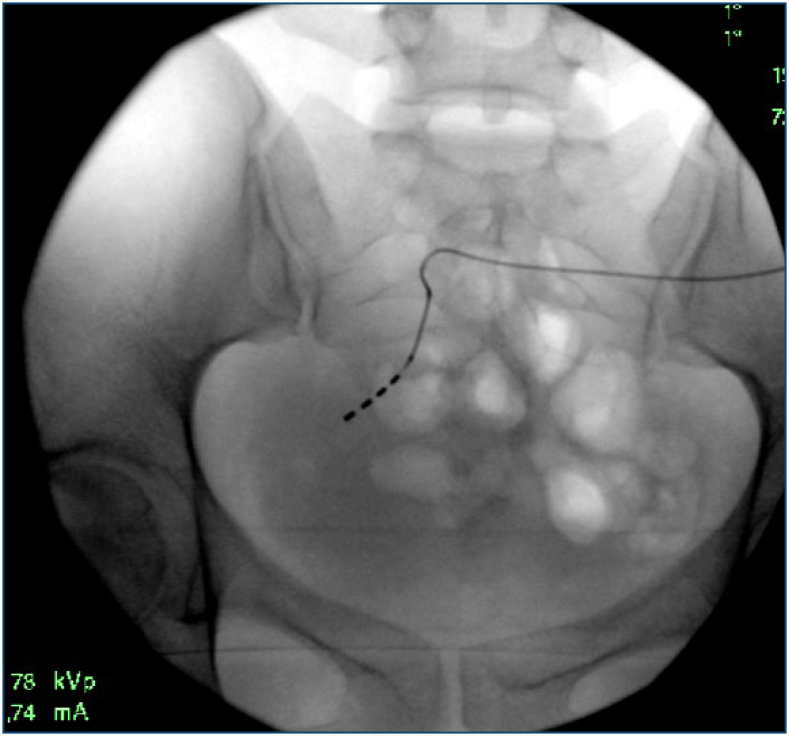
The placement of the sacral neuromodulation, SNM, electrode.

The surgery—inserting a temporary SNM electrode into the left S3 root—took place 6 months after the sphincteroplasty (Figure 3), which was performed under general anesthesia without complications. She was instructed to take care of the external stimulator the following day. The stimulator parameters were set, and the patient was discharged. The testing phase lasted for 3 weeks, during which there was a significant improvement in the patient's continence. She had a stool one to two times a day and was controlled by will. We proceeded with the implantation of a permanent SNM stimulator (Medtronic InterStim II), performed under local anesthesia. In the postoperative period, the patient experienced no complications, with bowel movements twice daily and continuously lasting 10–15 min. She continues to be followed up in the colorectal clinic, with further rehabilitation planned.

## DISCUSSION

The only rational solution for Hirschsprung's disease is currently surgical therapy^1^. In some cases, regardless of the chosen method, an imperfect technique can damage the dentate line and sphincter complex. Consequently, long-term disruption of stool formation and perception of basic sensations necessary for initiating bowel movements is observed. According to the preoperative examination of our patient, the anastomosis appears to be almost colocolonic. The part of the intestine responsible for continence (rectal ampulla and anal canal) was resected as part of the aganglionic bowel, leading to poor stool detection and possible FI. Childbirth injuries from grade 3 and above are a severe condition with a risk of long-term damage to her. Local anesthesia is sufficient at most for superficial injuries of grade 3a; for higher grades, general or regional anesthesia is preferred, allowing muscle relaxation to mobilize the retracted external anal sphincter (EAS). In cases of incomplete disruption of the EAS, the end-to-end method is chosen, where, after mobilizing the muscle, its edges are approximated with two to three mattress sutures, including the muscle fascia. For complete injuries, overlap sphincteroplasty is recommended, requiring more excellent dissection and mobilization of the muscle to overlap one end of the muscle over the other. For this, two to three individual long-term absorbable monofilament sutures are used. This technique is also recommended for redo-surgery for sphincter treatment, as non-absorbable sutures may cause discomfort and inflammatory complications^13^.

Treatment of OASIS should be carried out by adequately trained personnel. If such a team is unavailable, the suture can be delayed for 8–12 h without worsening the patient's prognosis. A single dose of second-generation cephalosporin is recommended for perioperative administration to reduce wound-healing complications. In the postoperative period, laxatives (such as lactulose) are also administered to reduce pain during the first defecation. Laxatives or bulk-forming agents are not recommended^14^.

In the present case, the determination of the degree of injury and the method of treatment are debatable. According to the birth protocol, there was a rupture of the perineum classified as grade IIIa. Still, during the first proctological examination, a palpable, complete lesion of the sphincter was found, confirmed by further examinations, suggesting a more likely grade IIIb or IIIc. In retrospect, evaluating the situation is inappropriate, and the authors of this article are not competent to do so either. However, it could be said that the degree of injury was underestimated, leading to the subsequent treatment approach.

The two primary techniques of delayed reconstruction of the anal sphincter are (i) end-to-end suture and (ii) suture with overlapping ends by 1.5–2 cm. Gynecologists prefer the first one, but anorectal surgeons more often choose to overlap a plasty^15^. Based on objective examination indices and patients’ subjective perceptions, some studies suggest the overlap technique is better^16^, while others do not^17^. We use the overlap plasty in our ward, as in the presented case. Despite that, the sphincter plasticity was loosening and new development of incontinence, which was likely compounded by the above-mentioned poorer sensitivity of the neorectum mucosa after the Swenson operation. The initial treatment for secondary FI after postpartum trauma to the anal sphincter is most commonly overlap plasty. Unfortunately, sphincter defect reconstructions are not as durable as previously expected. Long-term evaluations have revealed success rates ranging from only 35 to 50%. Sacral nerve stimulation for FI is an effective method with long-lasting effects. Some studies even describe treatment success with a defect in the external sphincter of up to 120°^18^. We decided to apply SNM treatment to exhaust conservative and surgical methods. The surgical procedure was performed without complications, and the treatment effect persisted for 3 months. She will continue to be monitored in the colorectal^19^ clinic for the long term. A surprising result for us was the reduction in stool frequency to two to three times a day from the long-term 10 after surgery for Hirschsprung's disease in childhood. SNM might enhance the signal from the damaged area of the rectal dentate line. Therefore, this area is open for further investigation and potential research.

## CONCLUSION

Hirschsprung's disease is characterized by the absence of the autonomic enteric system in varying lengths of the digestive tract. The rational solution for Hirschsprung's disease is currently surgical therapy, with the modified Swenson's operation being the method of choice. Because of technically imperfect execution, long-term issues with stool formation and perception of basic sensations necessary for triggering defecation might be observed.

Despite surgical intervention for postpartum sphincter trauma, some have residual sphincter defects, and symptoms of anal incontinence may appear either immediately after childbirth or with a delay. In such cases, sphincteroplasty using the overlap is appropriate. SNM is a successful method for smaller defects in the sphincter circumference. SNM might attenuate the stool frequency following surgery for Hirschsprung's disease.
