# Erratum

**DOI:** 10.1590/1806-9282.20240827ERRATUM

**Published:** 2024-11-11

In the manuscript "Hirschprung's disease and postpartum trauma leading to fecal incontinence: Why? How?", https://doi.org/10.1590/1806-9282.20240827, published in the Rev Assoc Med Bras. 2024;70(9):e20240827:

On page 2, Figure 1

**Where it reads:**

Figure 1. An intraoperative photography; the overlap plastic procedure.

**It should read:**

Figure 1. A 3D manometric model of the sphincter.

On page 3, Figure 2

**Where it reads:**

Figure 2. A 3D manometric model of the sphincter.

**It should read:**

Figure 2. An intraoperative photography; the overlap plastic procedure.

